# Megaureter

**DOI:** 10.1100/tsw.2010.54

**Published:** 2010-04-13

**Authors:** Steve J. Hodges, David Werle, Gordon McLorie, Anthony Atala

**Affiliations:** Department of Urology, Wake Forest University School of Medicine, Winston-Salem, NC, USA

**Keywords:** megaureter, hydronephrosis

## Abstract

Almost one-quarter of the children referred to a pediatric urologist for obstructive uropathy suffer from an obstructive megaureter. However, not all megaureters are due to obstruction, as some may be the result of reflux and many simply represent a slightly skewed stage of development that can result in a normal urinary tract if observed. As the use of fetal ultrasonography has expanded, the majority of children with megaureters are now diagnosed early in their development, and physicians are faced with the complex task of distinguishing which children need medical intervention and which do not. The surgical treatments of megaureter are well established, relatively simple, and effective if performed in the correct candidates. Therefore, research efforts in this field have recently focused on improving our ability to diagnose clinically relevant obstructive uropathy and examining the developmental causes of megaureter, and how this disorder may be prevented.

The term “megaureter” is simply a descriptive term about the diameter of the ureteral lumen. It means exactly what it says, “large” ureter. It implies no description of function of the ureter in terms of obstruction or reflux. A megaureter on imaging studies can be quite impressive, and of concern to physicians and parents alike. And, since the surgical techniques to repair megaureters are relatively simple and successful, it is tempting to try to improve surgically abnormal appearing ureters in the presence of marked dilation; however, the data have shown us that surgical intervention even in the setting of some pathology, such as obstruction, is not always warranted. Indeed, the major challenge in the management of megaureters is the clinical decision to intervene or not[1,2,3].

A ureter is considered a megaureter if the lumen is dilated. Although the dilation is rarely subtle, for the terms of definition any diameter larger than 8 mm is considered abnormal. Megaureters can be classified as obstructed, refluxing, obstructed and refluxing, or neither obstructing nor refluxing, using the international classification of Smith et al.[4]. Typically, if there is an obvious cause for the enlarged ureter, such as elevated bladder pressure due to a neuropathy, the term megaureter is not used. Rather, it is reserved for cases of hydroureteronephrosis and otherwise normal bladder and outlet function. The pathology of the ureter can also be primary (or intrinsic) or secondary (due to another urinary tract pathology). It is important to distinguish between cases of primary or secondary causes of the pathology, as in the case of secondary causes, the treatment is directed at the initiating pathology and not at the ureter[1,2,3,5].

The question of obstruction is a difficult one in cases of megaureter, much as in the children with ureteropelvic junction (UPJ) obstruction. Indeed, any megaureter labeled "obstructed" has, by definition, a delay in drainage of radiotracer on nuclear medicine renogram beyond the normal t½ of 20 min commonly used as a cutoff for urinary tract blockage. However, as Koff and Campbell described years ago, the true definition of obstruction lies not in the t½, but in the determination of the degree of obstruction that will lead to renal injury if it is not relieved[6]. This definition has proven difficult, and the inability to determine whether obstruction is clinically significant or not has led many a clinician to intervene for better or worse.

## INCIDENCE

Megaureter is a common diagnosis in children referred to a pediatric urologist for urologic evaluation, representing 23% of children with urinary tract obstruction. The diagnosis is more common in boys than girls, and more often is on the left side. It can be bilateral in 25% of cases, and the contralateral kidney is absent or dysplastic in 10–15% of cases. There is no clear genetic pattern of inheritance, although some cases do appear to run in families. Rarely, a UPJ obstruction will be present in conjunction with a ureterovesical junction (UVJ) obstruction in cases of megaureter[1,2,3].

Most cases of megaureter are now first detected with prenatal ultrasound and then diagnosed after birth. Some cases present clinically during childhood with abdominal pain, hematuria, and/or urinary tract infections. Megaureter may also be incidentally discovered later in life on imaging studies. It rarely leads to renal insufficiency[1,2,3].

## EMBRYOLOGY AND PATHOPHYSIOLOGY

There have been many elegant studies performed to describe the histologic appearance of megaureters and although they often differ, the common finding is an abundance of connective tissue in the abnormal ureter[7,8,9,10]. In fact, Lee et al. demonstrated that the collagen to smooth muscle ratio in normal ureters is 0.52, while it is 0.78 and 1.99 in obstructed and refluxing megaureters, respectively[11]. Other studies have demonstrated evidence of smooth muscle cells in these ureters that produce an abnormally elevated amount of collagen. The muscles in these ureteral segments have also been shown to respond abnormally to neurotransmitters, emphasizing the anomalous behavior of these cells[7,8,9,10].

### Primary Obstructive Megaureter

Primary obstructive megaureter is considered a functional obstruction. There is thought to be an aperistaltic juxtavesical (adynamic) segment in the ureter, leading to a lack of propagation of the ureteral peristalsis and therefore urine flow. This distal segment has been examined histologically and has been found to contain increased levels of collagen type I and III (predominantly type I). It is this increased fibrosis that is implicated in the disruption of intercellular communications and leads to uretero arrhythmias and obstruction[7,8,9,11].

There are many other theories regarding the development of obstructive megaureters, however. Some scientists have shown evidence of atrophy of the inner longitudinal muscles in these ureteral segments (the longitudinal muscles are the ones that transmit peristalsis) and hypertrophy of outer, compressive circular muscle, leading to obstruction[12,13].

The fact that so many obstructive megaureters resolve and develop into normal collecting systems over time has pushed many to define a maturational cause of obstructive megaureters, signifying that perhaps the renal urine production began slightly prematurely, before the ureter fully cannulated at its caudal end, leading to hydroureter. The full canalization of the mature ureter could then explain the resolution of the obstructive appearance of the ureter. Another maturational theory is that the obstruction represents a developmental evolution of the distal ureter from a single, circular muscle layer to the double layer (circular and longitudinal) of the child[1,3].

Other histologic findings claiming to display the causative aspect of the obstructive megaureter include distal ureteral segments with no muscle tissue present, but simply a fibrotic, static terminal end. Yet others have documented distal ureteral segments with a nonureteral, nondetrusor muscle that is excessively responsive to nonadrenergic stimulus, leading to almost tonic contraction[1,14,15,16].

Interestingly, the proximal, dilated ureteral segment has also been found to be composed of altered connective tissue, and this fibrosis and the dilation itself can lead to uretero arrhythmias and poor peristaltic wave transmission. It is important to note that the upper tract dilation (while appearing to be a significant pathology in and of itself) does play an important role in the urinary tract response to the presence of obstruction. The infant collecting system is more pliable than in more mature patients and this dilation allows for the dampening of pressure, allowing the kidneys to produce urine into a collecting system at close to physiologic pressures[1,2,3].

Other than the adynamic segment described above at the terminal ureter in the obstructive megaureter, other anatomic causes can lead to a similar clinical scenario. Both congenital distal ureteral strictures and distal ureteral valves can be almost indistinguishable from the classic obstructive megaureter[17,18,19].

## Secondary Obstructive Megaureter

Secondary obstructive megaureter represents an obstructive process secondary to elevated intravesical pressure of some other cause. Common causes include spinal dysraphism and neurogenic bladder, which may elevate detrusor pressure to over 40 cm $H_2O$, causing a physiologic obstruction and hydronephrosis in the collecting system. Non-neurogenic voiding dysfunction, if severe enough to elevate bladder pressure above the safe range, may also be a cause. Posterior urethral valves, or other causes of infravesical obstruction, can also lead to similar findings[1,18,19].

Other anatomic causes of secondary, distal ureteral obstruction include ureterocele, ectopic ureter, bladder diverticula, periureteral fibrosis, and external compression by retroperitoneal tumor, masses, or aberrant vessels[1,18,19].

## Primary and Secondary Refluxing Megaureter

Refluxing megaureters simply represent a refluxing ureter that happens to be dilated. The pathology mimics that of any refluxing ureter, with a short intravesical ureter and submucosal tunnel. They may be associated with abnormalities of the UVJ, making reflux more likely, such as periureteral diverticula. Some children present with megacystis megaureter syndrome, in which the bladder is markedly distended and thin walled, in addition to the ureters[18].

The distal segment of refluxing megaureters also shows histologic derangement with increased fibrosis (much like the obstructive megaureters); however, in these cases, the predominant collagen is collagen type III[11].

Refluxing megaureters can be a characteristic of Prune Belly syndrome as well. These ureters also often demonstrate increased collagen deposition distally, with the clinical manifestation ranging from inefficient peristalsis to distal obstruction[19].

## Refluxing Obstructed Megaureter

In refluxing megaureters, 2% also present with some degree of obstruction.

Although not intuitively apparent, refluxing ureters may lead to obstruction when the distal ureter that fails to coapt (reflux) also does not transmit peristalsis (obstruction). Alternatively, the ureter may have an ectopic insertion at the bladder neck, which refluxes when relaxed and obstructs when tightened[20].

### Primary Nonobstructive, Nonrefluxing Megaureter

Most cases of megaureter end up being of the nonobstructive, nonrefluxing variety. This is very heartening, as it confirms that simple observation will serve as the therapy for most children. However, as mentioned, the lack of obstruction can be difficult to prove[1,18].

Certain important points should be kept in mind when evaluating a megaureter that may help to prevent unnecessary intervention. First of all, the fact that an infant is born with a functioning kidney provides evidence that any degree of ureteral obstruction is not complete, as the kidney would not have formed normally in the setting of early or very high-grade obstruction. Also, as touched on earlier, the complex orchestration of embryologic development may have many variations that create an appearance of anomalous development, only to improve as the necessary steps of development are completed. The fetus makes larger volumes of urine compared to the infant, and if this diuresis precedes the natural canalization of the distal ureter, a megaureter may develop (maturational delay hypothesis). Since the ureter in the fetus is so compliant, small increases in urine flow can induce a ureteral dilation, even in the absence of obstruction and reflux. It is this compliant collecting system that allows the infant kidney to continue to function in the setting of varying degrees of obstruction or reflux without suffering pressure injury, so dilation may be beneficial and not harm the child, and is therefore not necessarily an indication for repair[1,2,3,18].

### Secondary Nonobstructive, Nonrefluxing Megaureter

The cases of nonobstructive and nonrefluxing megaureter due to a cause unrelated to ureteral anatomy are termed secondary. It is in this category that dilation due to high fetal urine output, increased compliance of fetal ureter (due to extracellular matrix composition, including elevated collagen type II, and elastin concentration), or a partial or transient obstruction during development (such as ureteral folds or delays in the development of normal peristalsis) occur[18].

There are many other relatively benign causes of secondary megaureter. For example, urinary tract infections can lead to temporary ureteral dilation due to the presence of bacterial endotoxins that can inhibit peristalsis. As mentioned, any increase in urine output can cause dilation of the fetal/infant collecting system. Some possible causes of diuresis include lithium toxicity, diabetes insipidus or mellitus, sickle cell nephropathy, or psychogenic polydipsia[18].

## DIAGNOSIS

In the current state of standard prenatal care and assessment, the widespread use of prenatal ultrasound has increased the prenatal diagnosis of megaureter. In fact, the majority of cases are now diagnosed prenatally. Cases detected later in life often present with urinary tract infection, hematuria, and/or pain[21,22].

Once diagnosed (prenatally or after birth), the first urologic evaluation is a complete renal and bladder ultrasound (Figs. 1 and 2). Ultrasonography is a simple, safe, and painless study that can provide important information on renal size, parenchymal thickness, echogenicity, and architecture, as well as renal pelvis and ureteral dilation, and bladder wall thickness, and even the urethra in some cases of urethral obstruction. Although an experienced urologist can infer certain functional diagnoses from ultrasound studies, it is important to remember that grey-scale ultrasonography is only descriptive and provides no details on renal function or drainage[19,21].

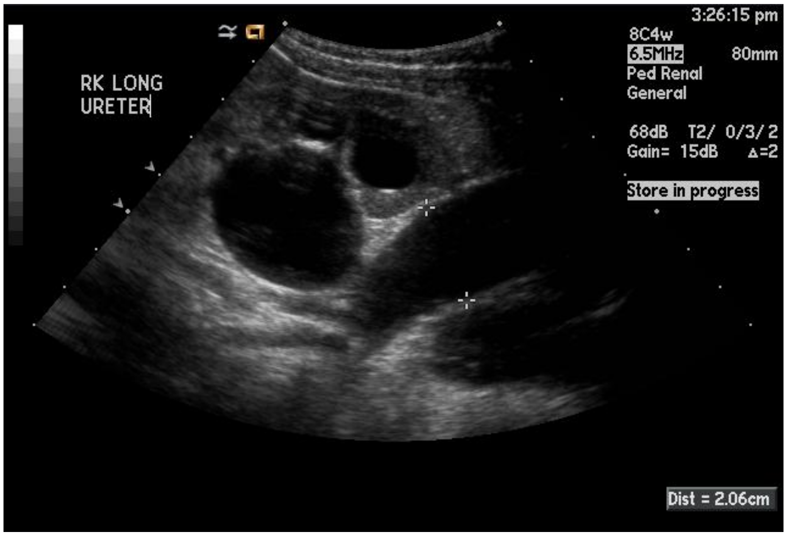

**FIGURE 1.** Ultrasound from obstructed megaureter demonstrating dilated collecting system and proximal ureter.

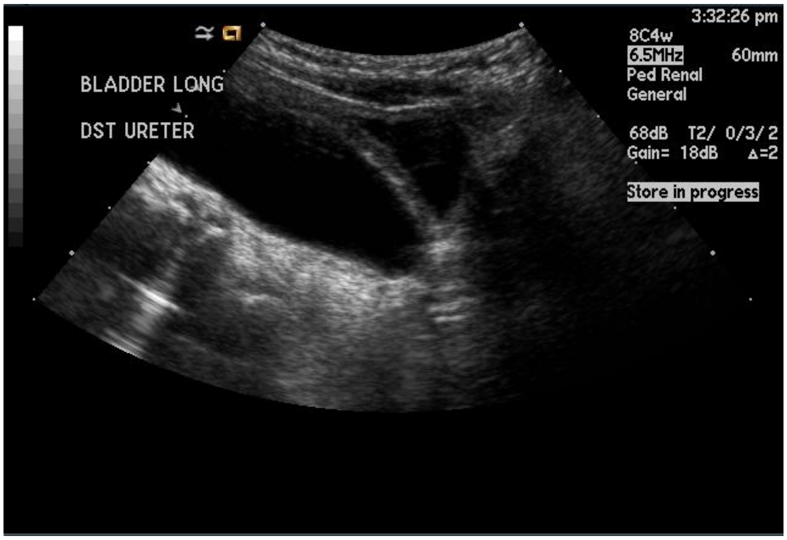

**FIGURE 2.** Ultrasound from obstructed megaureter demonstrating dilated distal ureter to tapered distal segment.

If renal pelvis or ureteral dilation is observed on ultrasonography, a voiding cystourethrogram is needed to rule out reflux (Fig. 3). This also allows for the complete anatomic evaluation of the bladder and urethra[18,19,21].

The most commonly used tool presently used for the evaluation of obstructive nephropathy is the diuretic renogram (Fig. 4). The two radiotracers useful for the evaluation of obstruction include DTPA (freely filtered by the kidney, and neither secreted nor reabsorbed) or Mag3 (filtered and secreted by the renal tubules). Mag3 has become the standard agent for use in infants as it provides improved imaging in poorly functioning systems[3,18].

Unfortunately, there are many variables involved in diuretic renography that can influence study findings and many are user dependent. Some of these variables include tracer dosing, timing of diuretic administration, patient hydration, and determination of the study areas of interest[1].

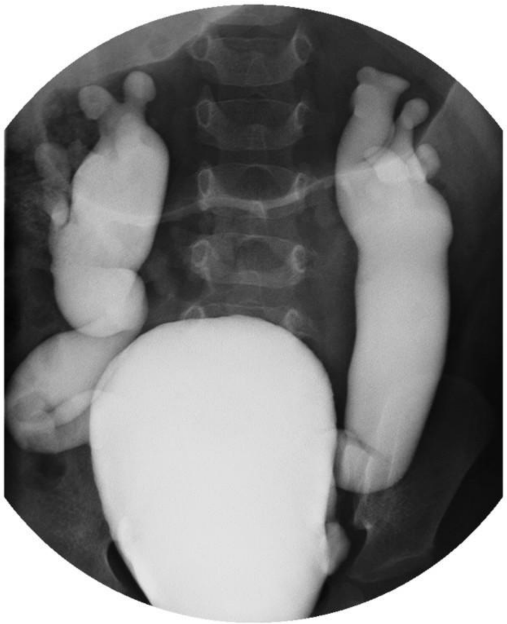

**FIGURE 3.** Voiding cystourethrogram demonstrating megacystis megaureter, with bilateral high-grade reflux.

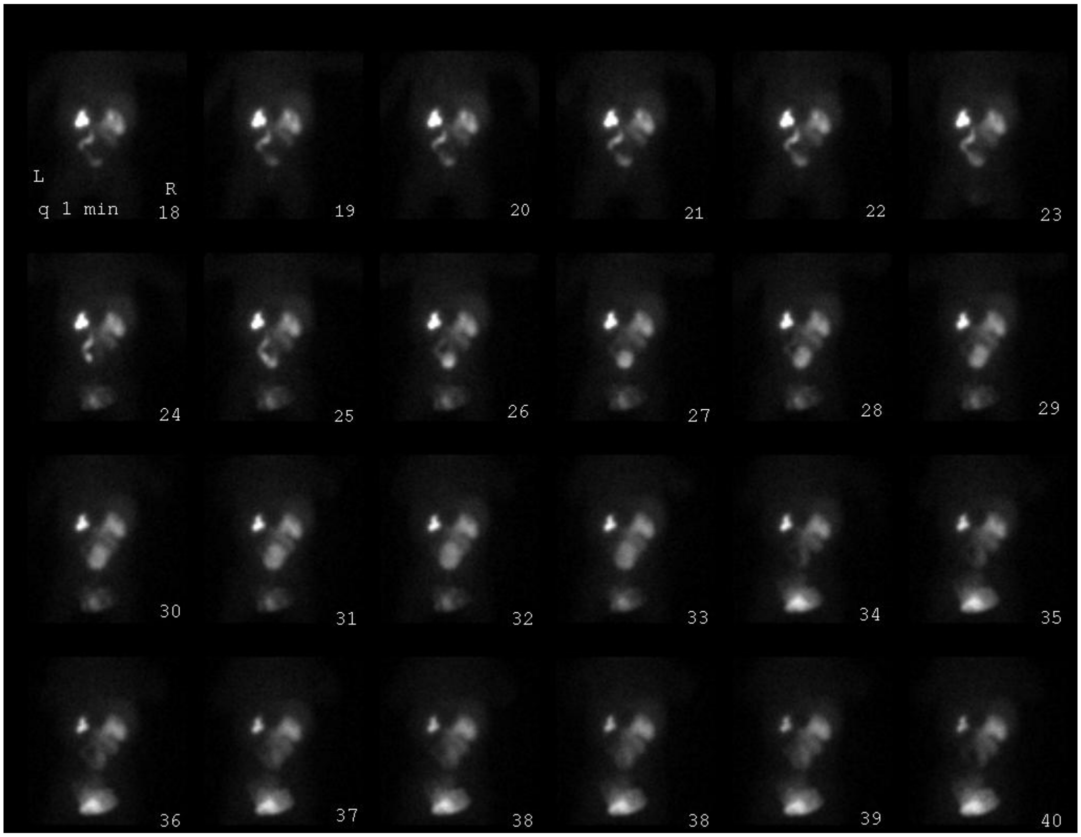

**FIGURE 4**. MAG-3 diuretic renogram demonstrating accumulation and stasis of urine in dilated ureter.

False-positive studies for obstruction are possible if diuretic renography is performed in children under 3 months of age, as the renal tubules of newborns show blunted response to diuretics and these children have low baseline glomerular filtration rates. Other causes of false-positive studies can include dehydration or a single kidney glomerular filtration rate of <15 ml/min[1,18].

In order to ensure that renogram studies are preformed in a standard fashion, a well-tempered renogram has been described. The well-tempered renogram has three important components: (1) 10–15 ml/kg crystalloid hydration prior to the study, (2) 1 mg/kg of Lasix administered at the peak of tracer accumulation in the kidneys (plateau), and (3) a catheter in place during the entire study. It is important that all these parameters are controlled in order to provide the best information from these studies[18,21].

As difficult as it is to perform a correct diuretic renogram, the interpretation of the study is even more challenging. All urologists are familiar with the range of t½ values associated with obstructed or unobstructed collecting systems (<10 min unobstructed, 10–20 min equivocal, >20 min obstructed). However, in cases of megaureter, the dilated collecting system can have such a large capacity that the drainage of the radiotracer is delayed despite the absence of true obstruction[18,21].

To compensate for the equivocal data provided by drainage times in renograms performed in children with dilated collecting systems (even with diuretic use), physicians have developed other tools to improve the function of renography in this setting. For example, the fractional uptake of the radiotracer should be equal in two unobstructed kidneys, and a difference in this parameter can be more important than drainage in dilated systems. Despite this extra information, renography can still be difficult to interpret[1].

Other attempts to improve renography include the F-15 method, in which Lasix is given 15 min before tracer dosing. This method is thought to decrease false-positive studies in children with dilated or poorly functioning systems, as it coordinates the diuretic activity to the timing of tracer administration[1].

The extraction factor is another attempt to improve renographic interpretation. The extraction factor is an estimate of single kidney function with DTPA and is the percentage of tracer uptake 2–3 min after injection. The normal extraction factor is 1.5% in newborns and 2.5% by the first year, and it can be used by correlating it to the glomerular filtration rate by the factor 0.92, or observing for changes in relative renal function over time[1].

Historically, a Whitaker's test was used in cases of hydronephrosis to rule out obstruction using pressure/flow measurement; however, these are rarely used currently due to the invasive nature of the procedure[18].

Physicians from Atlanta have demonstrated that magnetic resonance urography can be as effective as renal scintigraphy in detecting renal obstruction and calculating an estimate of glomerular filtration rate, but with much improved anatomic detail. These studies require expertise to perform, however, which may not be available in all centers, and requires at least some degree of child sedation, limiting its widespread use[23].

Modifications to ultrasonographic studies have also been attempted in an effort to improve their diagnostic utility. For instance, the resistive indices calculated from Doppler ultrasonography can correlate with obstruction, with resistive indices of >0.70 considered normal by the first year of life (but truly, these resistive indices were described originally in adult kidneys and extrapolation of these data to infants is controversial). Doppler ultrasound studies can be improved with fluid bolus or Lasix administration because the diuresis forces the resistive indices lower in unobstructed kidneys and higher in obstructed kidneys. In the setting of a hydronephrotic kidney and a contralateral normal kidney, calculating the difference in resistive indices can be helpful, with differences greater than 0.06–19.1 considered obstructed[1].

## TREATMENT

As mentioned several times in this review, the treatment of megaureters is not difficult or particularly controversial; the decision to treat is where the art lies, in defining obstructed megaureters from nonrefluxing, nonobstructed variants. This may not always be possible. Fortunately, most prenatally detected cases are asymptomatic and may simply be observed.

## Primary Refluxing Megaureter

All urologists are familiar with the standard treatment of reflux and the treatment of primary refluxing megaureter is no different. Initially, even with severe dilation and high-grade reflux, medical management (antibiotic prophylaxis) and observation are all that is necessary. Surgery is only considered for persistent high-grade reflux in older children (especially with recurrent pyelonephritis) and in infants that have failed medical management. As the complication rate for ureteroneocystostomy is high when performed in children under a year of age, cutaneous ureterostomy or vesicostomy may be used as a temporizing measure in infants requiring surgical intervention[18].

## Secondary Refluxing or Obstructive Megaureter

Obviously, secondary reflux must be treated by addressing the cause of elevated intravesical pressure leading to reflux. For example, in children with posterior urethral valves and reflux, often valve ablation and proper bladder management will lead to a rapid resolution of the reflux. Neurogenic bladders with elevated detrusor leak point pressure (>40 cm $H_2O$) must be treated with a combination of medical therapy (i.e., anticholinergic medication), clean intermittent catheterization, and surgery, if necessary. Often cases of Prune Belly and diabetes insipidus can be managed with observation, presuming the appropriate medical therapy is initiated[18].

## Nonobstructive vs. Obstructive Megaureter

In the cases of possibly obstructed megaureters, the decision to intervene surgically is a difficult one. Even in cases of obvious obstruction, early surgical intervention is fraught with a higher complication rate. The basic tenet that should be followed is that no surgery should be performed as long as renal function is not significantly affected and urinary tract infections are not a major issue. Instead, antibiotic suppression with close observation is all that is required. Typically, surgical repair is warranted between 1 and 2 years of age if the condition is worsening[1,3,18].

In certain rare cases, early intervention is necessary. In order to prevent the complications associated with nonrefluxing, reimplant surgery in infants, other surgical options should be considered, such as loop ureterostomy, refluxing reimplant, and even ureteral stent placement. Definitive repair in infants should only be performed by experienced hands[1,3,18].

In terms of forming algorithms to decide which children will require surgery, no good parameters dictate the children that will resolve and those that will worsen. In general, over 70% of cases resolve over 2 years of follow-up. While there is no correlation with any definable factors (such as degree of hydronephrosis) with regard to which children will require surgery and which will not, there is a correlation of age of resolution and grade of dilation in infants[24].

## Surgical Techniques

The surgical techniques used for the definitive treatment of refluxing and obstructive megaureters both involve ureteral reimplantation of the dilated ureter. The same parameters used to ensure successful surgery as traditional reimplant surgery apply to megaureters as well (i.e., 5:1 tunnel length to ureter diameter ratio). In cases of obstructive megaureters, the distal adynamic segment must be completely amputated from the ureter (Fig. 5), and often once the obstruction has been relieved, the ureteral diameter decreases to a size that allows for standard reimplant without tapering. Most refluxing and obstructive megaureters do require tapering, however, to allow for a submucosal tunnel size that will fit the pediatric bladder[2,3,18].

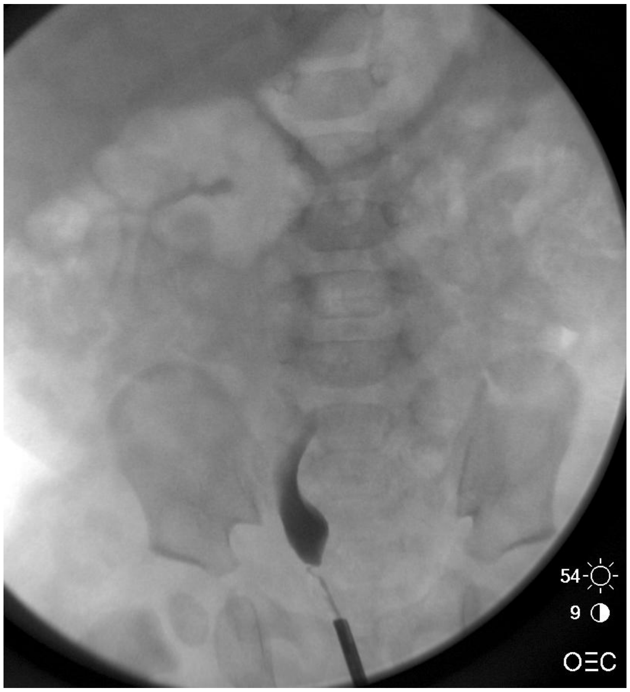

**FIGURE 5.** Retrograde pyelogram of adynamic distal segment in obstructive megaureter.

There are three main types of ureteral tailoring techniques, including ureteral plication or infolding for moderately dilated ureters, and excisional tapering for massively dilated or thickened ureters. Excisional tapering, described by Hendren, is easily performed with the use of Hendren clamps, allowing for exact tapering of the ureter to the appropriate size, although critics argue that even when the medial ureteral blood supply is maintained by excising the lateral ureter, there is a high risk of ischemia to the distal ureter with this procedure. To mitigate the risk of ureteral ischemia, plication techniques, such as the Starr and Kaliscinski placation, have been developed and have demonstrated excellent results. The most common serious complications from tapered reimplants include persistent reflux and ureteral obstruction[21,25,26,27].

In instances of duplicated collecting systems, in which only one of the moieties is massively dilated, then ipsilateral ureteroureterostomy or ureteropyelostomy may easily be performed to relive obstruction or reflux. Reimplant of both ureters of a duplicated system must maintain the common sheath to preserve ureteral blood supply[18].

## CONCLUSION

Although the therapies for megaureters are successful and safe, the inability of a urologist to evaluate neonatal urinary tract obstruction accurately can make clinical decision making in cases of megaureters difficult. There has been great progress in the field, however, as the move away from immediate surgical therapy for these children (much as in the case of congenital UPJ obstruction) has saved many children from unnecessary surgery. As diagnostic tools improve, and developmental science gains greater understanding of the causes and reasons for megaureter development, hopefully only children absolutely requiring intervention will be exposed to its risk, and perhaps disease prevention will be possible.
